# Spatial distribution and hydrogeochemical evaluations of groundwater and its suitability for drinking and irrigation purposes in kaligonj upazila of satkhira district of Bangladesh

**DOI:** 10.1016/j.heliyon.2024.e27857

**Published:** 2024-03-23

**Authors:** A.H.M. Shofiul Islam Molla Jamal, Nisat Taslum Jhumur, Md Aftab Ali Shaikh, Mohammad Moniruzzaman, Md Ripaj Uddin, Md Abu Bakar Siddique, Muhammad Abdullah Al-Mansur, Md Ahedul Akbor, Jahan Tajnin, Sharmin Ahmed, Rashed Mahmud

**Affiliations:** a Institute of National Analytical Research and Service (INARS), Bangladesh Council of Scientific and Industrial Research (BCSIR), Dhanmondi, Dhaka 1205, Bangladesh; b Department of Chemistry, University of Dhaka, Dhaka 1000, Bangladesh; c Bangladesh Council of Scientific and Industrial Research (BCSIR), Dhanmondi, Dhaka 1205, Bangladesh; d Central Analytical and Research Facilities (CARF), Bangladesh Council of Scientific and Industrial Research (BCSIR), Dhanmondi, Dhaka 1205, Bangladesh

**Keywords:** Hydrochemistry, Salinity, Multivariate statistical analysis, Irrigation suitability, Water quality index, Spatial distribution

## Abstract

Groundwater is a significant water resource for drinking and irrigation in Satkhira district, Bangladesh. The depletion of groundwater resources and deterioration in its quality are the results of the confluence of factors such as industrialization, intensive irrigation, and rapid population growth. For this reason, this study focused on the evaluation of tubewell water of six unions of Kaligonj upazila in Satkhira district, which is situated in the coastal southwest part of Bangladesh. Major and trace elemental concentrations were assimilated into positive matrix factorization (PMF) to identify potential sources and their respective contributions. Principal component analysis (PCA) revealed that groundwater salinization and manmade activities were the primary causes of heavy metals in the coastal groundwater. Its average pH value was found to be 7.5, while Dissolved oxygen, Total dissolved solids, salinity, and conductivity, with values ranging from 1.18 to 7.38 mg/L, 0.5–4.88 g/L, 0.4–5%, and 0.95 to 8.56 mS/cm, respectively. The total hardness average value was 561.7 mg/L, classified into the very hard water categories, which is why 90% of the tubewell water samples were unfit for household purposes. All samples had an excessive level of arsenic present. The iron concentration of fifteen (15) samples crossed the standard limit according to WHO 2011 value. Around 63% of the samples were of the Na^+^-K^+^-Cl^-^-SO_4_^2-^ type, and about 72% were sodium-potassium and alkali types. 98% of samples were covered in chloride and bicarbonate. The findings showed that 45.83% of the groundwater samples had negative Chloroalkaline index (CAIs), while 54.16% had positive. The permeability index (PI) was an average of 73%, and residual sodium carbonate (RSC) averaged 260.2 mg/L, and the findings clearly showed that 80% of the samples weren't appropriate for irrigation. According to the sodium adsorption ratio (SAR) value, 65% of the samples fell into the unsuitable category. These calculations indicated a high overall salinity hazard in the study area, which may be caused by the intrusion of sea water given that the study area is close to the coastal region. Findings compared to standards revealed that the majority of the samples were deemed unfit for drinking and irrigation purposes. Hence, additional attention must be paid to this area to ensure the availability of drinkable water and to preserve sustainable farming practices.

## Highlights

• Salinity Intrusion occurred in the groundwater of Kaligonj upazila.
• The water quality of the studied areas is not suitable for irrigation and drinking purposes.
• Groundwater salinization and anthropogenic activities were two potential sources identified.

## Graphical abstract

**Figure:**
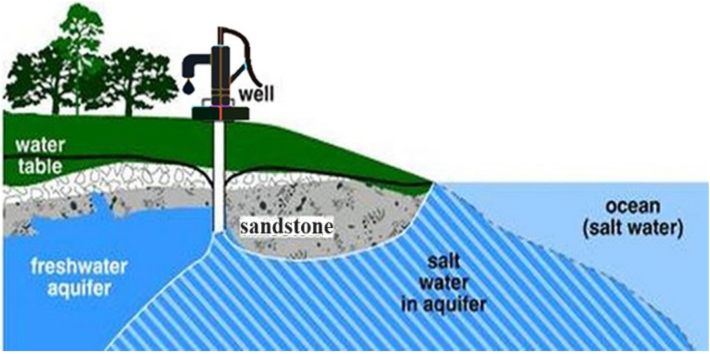

## 1 Introduction

Groundwater is a significant water resource that plays a crucial role in regional development, particularly in areas with limited water resources such as coastal regions, arid regions, and megacities [[1], [2], [3]]. Bangladesh is regarded as one of the world's most environmentally endangered countries. Its 710 km south coastline contains many locations extremely vulnerable to sea level rise. For many areas, especially the south-east, central [4] and southwest [[5], [6], [7]] parts of Bangladesh's coastal areas, water, and soil salinity are common threats. This saline water is used for drinking, irrigation, household, and fisheries [8]. Groundwater sources provide over 90% of the nation's drinking water [9] and 75% of its agricultural water in Bangladesh [10]. Shallow aquifers deteriorate due to high population pressure, excessive human activity, incorrect resource usage, and a lack of suitable management procedures. Aquifers in the research area are exposed to maritime influence due to strong anthropogenic pressure inside and outside the area. Bangladesh has three different types of groundwater aquifers [11]: the upper shallow unconfined aquifer, the middle confined aquifer, and the deep confined aquifer. Almost everywhere in the nation, people use the uppermost shallow aquifer to extract water for drinking and irrigation, even though the middle-confined aquifer is the primary aquifer [12]. Bangladesh's surface and groundwater systems face a number of water quality issues, particularly in the country's southwest coastal regions where salinity is currently a major concern [13,14]. Groundwater quality has been investigated by many researchers in different parts of the world [[15], [16], [17], [18], [19], [20], [21], [22], [23], [24], [25]]. In developing nations, issues pertaining to groundwater are particularly serious. With their vast populations, south Asian nations like Bangladesh, Pakistan, India, and others rely mostly on agriculture for their livelihood. Seawater intrusion in coastal aquifers is primarily caused by overexploitation of groundwater, sea level rise, prawn farming, and other factors that are causing concern globally. Groundwater pollution has resulted from overuse of groundwater and groundwater contamination due to unscientific irrigation techniques and a lack of technical expertise in groundwater extraction [26].

Almost 32% of Bangladesh's total area is in the shoreline areas [27,28]. Out of 64 districts with 140 upazilas, 19 are in Bangladesh's coastline region. Many researchers have conducted different studies about the coastal regions in Kaliganj Upazila, Satkhira district of Bangladesh. Shrimp farming is mostly what the locals in this area do for a living. Physicochemical parameters, nutrient content, bacterial contamination, and metal content of water and sediment samples were investigated in shrimp "Gher" (Farms) [29]. The water from various school tubewells was analyzed for arsenic, iron, and chloride in the Satkhira district [30]. The state of good aquaculture practices (GAP) at the culture stage in shrimp ghers in the Satkhira district's Shyamnagar and Kaliganj upazilas was assessed [31]. The safe water adaptability index was calculated [32] by considering the socioeconomic, institutional, physicochemical, and environmental viewpoints of the various upazilas in Satkhira. The shrimp pond has been the subject of the majority of this investigation. But there is hardly any research focusing on particularly groundwater hydrochemical properties and source apportionment of the Kaligonj area. As far as we know, no experiments have been conducted regarding the water quality in this region. Finding the source and distribution of various tubewell water constituents in the study area is a novel aspect of this research. The hydrochemical properties of groundwater and the distribution of sources in the Kaligonj region have received inadequate interest in research. This study will serve as an intellectual resource to counter the public health and agriculture sectors' susceptibilities in coastal Bangladesh and other parts of the world. By improving planning, implementing sustainable infrastructure, and providing adequate drinking and irrigation water, we can still protect the economies and communities in coastal areas.

Thus, the core objective of this study is (i) to create a water quality index that takes hydrochemical factors into account and to map out their spatiotemporal distribution and (ii) to evaluate probabilistic source apportionment in the groundwater of Bangladesh's coastal districts. The appropriateness of the coastal shallow groundwater for drinking and agricultural uses is evaluated using a thorough water quality investigation.

## 2 Materials and methods

### 2.1 Studied area

**Fig. 1:**
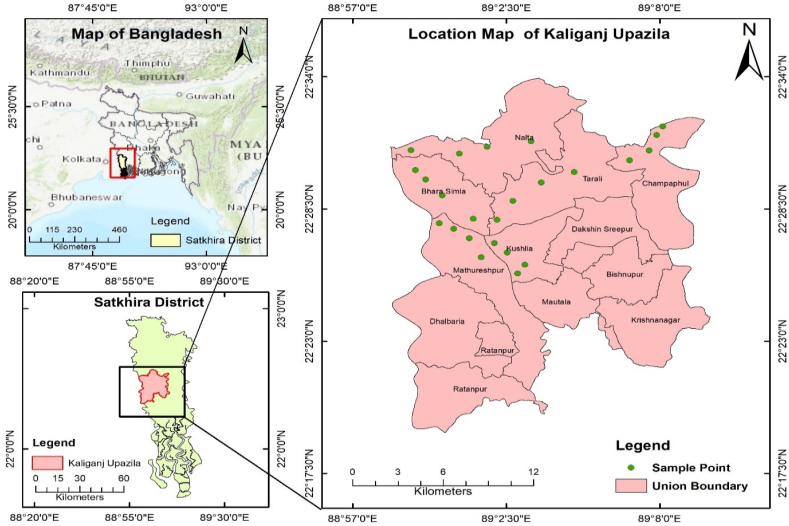
Sampling sites based on Geographic Information System (GIS).

Kaliganj, an upazila of Satkhira, is located (Fig. 1) between 22°17′ and 22°34′ N latitudes and between 88°57′ and 89°80′ E longitudes. It is 247 km from Dhaka and situated on the riverside Arpangachhia in the southwestern part of Bangladesh. It has an area of 333.79 sq. km. The studied region has a population of around 2.5 million people, with agriculture and fishing serving as the two main economic sectors. The study area is characterized by monsoonal climate with a tropical wet and dry or savanna climate. The southwest monsoon of the Indian Ocean controls this region's tropical climate and rainfall patterns. Around 1710 mm of rainfall occurs on the coast each year on average. Approximately 80% of the rainfall falls during the monsoon months, with almost no rain falling during the winter months, and the minimum and maximum temperatures are 12.5 and 35.5 °C, respectively [33]. The annual evaporation rate in the area is between 900 and 1500 mm. The evaporation rate exceeds the rainfall rate from November to May using decadal analyses of evaporation data [34]. The study area's land topography appears to be very flat, with land elevations never exceeding 5 m above mean sea level. The rivers Ganges, Brahmaputra, Meghna, and their tributaries and distributaries created Bangladesh's current floodplains [35]. The Ganges deltaic aquifer system in the entire part of southwestern Bangladesh was built primarily by late Holocene deposition to current Ganges River sediments [35]. The geological information of the current subsurface revealed that the majority of the country's good aquifers are found at a depth of 30–130 m, where sediments of mostly medium to fine sand, silt, and clay types were deposited [36]. The water in this aquifer is mostly saline in the coastal zone, with occasional pockets of freshwater [11]. The hydrology of the study area's coastal plain is governed by a complex interaction of fresh water flow from upstream, tides and tidal flows from the Bay of Bengal, tropical cyclones, storm surge, and other meteorological effects from the sea, and the physiography of the coastal plains [37]. Twenty-four (24) groundwater samples were taken in December 2022 from six unions in Kaliganj, Satkhira (Nalta, Mathureshpur, Bhara Simla, Tarali, Champaphul, and Kushlia) (Table 1).

**Table 1:** Sampling Locations of the studied area.

Sampling Stations	GPS value of the location		Sample ID
	Latitude	Longitude	
**Nalta**	22.51583	88.98471	STW-01
	22.51351	89.01363	STW-02
	22.51818	89.03031	STW-03
	22.52204	89.05642	STW-04
**Bhara Simla**	22.50196	88.98742	STW-05
	22.49552	88.99354	STW-06
	22.48451	89.00336	STW-07
	22.46829	89.02193	STW-08
**Tarali**	22.46755	89.03613	STW-09
	22.48076	89.04569	STW-10
	22.49337	89.06257	STW-11
	22.50063	89.08229	STW-12
**Champaphul**	22.50891	89.11531	STW-13
	22.52631	89.13138	STW-14
	22.51563	89.12728	STW-15
	22.53241	89.13512	STW-16
**Mathureshpur**	22.46517	89.00172	STW-17
	22.46134	89.01029	STW-18
	22.45483	89.01957	STW-19
	22.44161	89.02674	STW-20
**Kushlia**	22.45143	89.03452	STW-21
	22.44485	89.04228	STW-22
	22.43635	89.05276	STW-23
	22.43025	89.04842	STW-24

### 2.2 Sample preparation and analysis

The samples of water were taken using clean plastic bottles (500 mL). After pumping the tube well for ten (10) minutes, water samples were taken from each sampling place. The samples were obtained in triplicate using three distinct plastic bottles equipped with airtight caps [38]. For the purpose of analyzing trace elements, water samples were acidified with concentrated HNO_3_ (2 mL/L) during the sampling process to achieve a pH level below 2 for metal analysis. On the other hand, water samples were collected without acidification for the analysis of anions [39]. The samples were promptly labelled, sealed tightly with corks to maintain an airtight environment, transported to the laboratory as soon as feasible, and stored at a temperature of 4 °C [[39], [40], [41]]. The samples were examined for various hydrochemical characteristics [41]. The pH and Electrical Conductivity (EC) were measured during sampling using portable meters, the EcoScan Ion 6 and Hanna HI 8633, respectively. Each portable meter was calibrated following the equipment manuals using standard solutions. Total dissolved solids (TDS) were calculated by weighing the solid residue left after measuring the volume of water samples and evaporating them until they were dry [APHA, 2540C] [39].

Ion chromatography (SHIMADZU LC-20AD) was used to quantify the major anions fluoride (F^−^), nitrate (NO_3_^−^), sulfate (SO_4_^2−^), and chloride (Cl^−^) (APHA 4110B). Carbonate (CO_3_^2−^) and bicarbonate (HCO_3_^−^) were determined by a titrimetric technique using HCl (APHA, 2320 B). A flame emission spectrophotometer (Model SHIMADZU, AA-6401F) was used to measure potassium (K^+^) and sodium (Na^+^) [APHA, 3500-NaB, 3500-KB] [42]. Calcium (Ca) [APHA, 3110 B], magnesium (Mg) [APHA, 3111 B], manganese (Mn) [APHA, 3110 B], arsenic (As) [APHA, 3114.C], and iron (Fe) [APHA, 3110 B] were measured by an atomic absorption spectrophotometer (AAS, Models: AA240FS and SpectrAA220, Varian, Australia) [[43], [44], [45], [46]]. Physicochemical studies were performed at the Institute of National Analytical Research & Service (INARS, an ISO/IEC 17025:2017 accredited laboratory) of Bangladesh Council of Scientific and Industrial Research (BCSIR) in Dhaka, Bangladesh.

To guarantee the accuracy of the data, meticulous measures were taken during sample collection, preparation, and analysis to prevent any form of contamination. In order to obtain accurate data, we used high-quality concentrated HNO_3_ (Fluka Analytical, Sigma-Aldrich, Germany) and deionized water (EC: 0.2 μS cm^−1^ and resistance: 18.2 ΩM cm at 25 °C) for preparing samples and standards in elemental analysis. In order to minimize the spread of contaminants, all glassware underwent an overnight cleaning process using a 10% HNO_3_ solution, followed by multiple rinses with deionized water. The pipettes, volumetric flasks, and AAS instrument were calibrated by authorized sources as part of the maintenance of our ISO/IEC 17025:2017 accredited laboratory (INARS, BCSIR, Dhaka). For the analysis of anions, we used water samples that were not acidified. For chemical elements and anions, NIST (National Institutes of Standards and Technology, USA) traceable Certified Reference Materials (CRM) were utilized to generate calibration curves (linearity ≥0.99) and conduct quality checks on the measurements. The analysis's accuracy and precision were guaranteed by conducting replicated analyses of both CRM and samples. In order to validate the dependability of the instruments, the quality of the analysis was further assessed through the measurement of standard samples procured from an alternative source (Scharlau, Spain). The process of quality control involved the sequential analysis of the sample blank, method blank, and CRM subsequent to five samples. The final results were presented as the mean of triplicate measurements for each sample, with a relative standard deviation of less than 5%.

### 2.3 Drinking and irrigation suitability analysis

#### 2.3.1 Positive matrix factorization (PMF)

The PMF is a reliable multivariate factor-based model based on the least-squares method [47]. The PMF incorporates non-negativity requirements and independently evaluates each data point [48,49]. The source apportionment assessments of all samples were conducted using the US EPA PMF 5.0 model [50]. The input data matrix is partitioned into two matrices that consist of factor profiles. These factor profiles represent the concentration of species and the contribution of factors for each sample, as determined by the PMF method. The PMF mathematical formula is represented as Equation (1) and has been reported in multiple research studies [51,52].

(1) $$X_{\text{ij}}=\sum_{k=1}^{p}g_{\text{ik}}f_{\text{jk}}+e_{\text{ij}}$$

Where p represents the number of factors, $g_{\text{ik}}$ represents the contribution of the kth factor on the ith day, $f_{\text{jk}}$ represents the mass fraction of the jth compound from the kth source, and $e_{\text{ij}}$ represents the residual for each sample or species. $X_{\text{ij}}$ is the concentration value of the jth metal species on the ith day of sampling.

Q, the key PMF parameter, is introduced, and two variants of Q, Q (true) and Q (robust), are measured for conducting the model, which assesses the parameters' goodness-of-fit. It has been observed that the optimal solution is frequently identified by the lowest Q (robust) value along the path (i.e., minimal Q) where excluded sample points did not suit the model. After attaining a minimum Q value, the PMF model is able to generate unique source profiles and contribution data. The object function Q is reduced utilizing the PMF technique with modified g, f, and p values. In (Equation (2)), Q is defined as where $S_{ij}$ represents the degree of uncertainty for the jth species in the jth number of samples. PMF 5.0 functions by default in a robust mode that reduces the influence of outliers on the contributions and profiles' ability to fit. The concentration and uncertainty of the two data files serve as essential inputs for each sample in PMF modelling. Each element was pretreated and validated based on the chaotic or absent outliers and values below the method detection limit (MDL). If any outliers exist, they are omitted with caution. The species exhibiting concentrations below the method detection limit (MDL) were substituted with quantities equivalent to half of the MDL [[53], [54], [55]]. The uncertainty data file was generated using the empirical equation below (Equation (2)).

(2) $$\sigma_{ij}=0.01\;\left(X_{ij}+{\bar{X}}_{j}\right)$$

The variable $X_{ij}$ represents the observed concentration of metal, while ${\bar{X}}_{j}$ denotes the average value of each sample. Additionally, $\sigma_{ij}$ represents the estimated measurement error for the jth species in the ith sample. The factor 0.01 was determined through the utilization of trial-and-error analysis. The methodology mentioned above is employed to evaluate and quantify levels of uncertainty [56,57]. Therefore, the calculation of uncertainty Sij can be determined using equation (3) as follows:

(3) $$S_{ij}=\sigma_{ij}=C_{3}X_{ij}$$

$C_{3}$ represents a constant, while $\sigma_{ij}$ represents the projected measurement error described in Equation (3). Several additional studies estimated the uncertainty of variables when measurements or methodological data were available to estimate error [56,57].

#### 2.3.2 Chloroalkaline index

Understanding how groundwater's chemical composition varies over time and the subsurface flow pathways is crucial. By examining the chloroalkaline indices (CAIs) suggested by Ref. [58] it is possible to comprehend the alterations in groundwater's chemical composition due to the method in which it flows. He proposed the CAI 1 (Eq. (4)) and CAI 2 (Eq. (5)) chloroalkaline indices to understand the ion exchange mechanisms between shallow aquifers and its adjacent environment. The exchange calculated using the corresponding equations is evaluated using the CAIs.

(4) $$\text{Chloro}-\text{alkaline}\;\text{indices},\text{CAI}1=\left[{\text{Cl}}^{-}\;–\;\left({\text{Na}}^{+}+K^{+}\right)\right]/{\text{Cl}}^{-}$$

(5) $$\text{Chloro}-\text{alkaline}\;\text{indices},\text{CAI}2=\left[{\text{Cl}}^{-}\;-\;\left({\text{Na}}^{+}+K^{+}\right)\right]/\;\left({\text{SO}}_{4}^{2-}+{\text{HCO}}_{3}^{-}+{\text{CO}}_{3}^{2-}\right)$$

#### *2.3.3* Sodium percentage (Na^+^ %)

Because of its reactivity with soil and subsequent reduced soil permeability, sodium is a key ion used to categorize agriculture. Generally, the percentage of Na^+^ is used to determine if water is appropriate for agriculture (Wilcox, 1955). Equation (6) allows for the expression of Na^+^ as a percentage of sodium or soluble-sodium percentage (Na^+^%).

(6) $$\%{Na}^{+}=\left(\frac{{Na}^{+}+K^{+}}{{Ca}^{2+}+{Mg}^{2+}+K^{+}+{Na}^{+}}\right)\times 100$$

#### *2.3.4* Residual sodium carbonate (RSC)

The appropriateness of water for irrigation is influenced by the ratio of carbonate and bicarbonate to calcium and magnesium in water. By causing organic matter in the soil to dissolve and create a black stain on the surface of the soil when it dries, an excessive presence of sodium bicarbonate and carbonate affects the physical features of the soil [59,60]. This excess is known as RSC, and the formula determines it [61]. Equation (7) is presented,

(7) $$RSC=\left(\;{CO}_{3}^{2-}+{HCO}_{3}^{-}\right)-\left({Ca}^{2+}+{Mg}^{2+}\right)$$

#### *2.3.5* Sodium adsorption Ratio (SAR)

Sodium adsorption ratio (SAR), defined as sodium or alkali threat, is a crucial metric for assessing irrigation water quality. Inferior output results from higher salinity, which decreases plant osmotic activity and prevents water access to the aerial parts of plants, such as branches and leaves [62]. In addition, the use of irrigation water with elevated levels of sodium ions (Na^+^) and reduced levels of calcium ions (Ca^2+^) has been seen to facilitate ion exchange processes by reaching saturation levels of Na^+^ ions. Consequently, this leads to the disruption of soil structure due to the dispersion of clay particles [63]. This degradation of soil structure has been found to impede agricultural productivity due to the consequent challenges in cultivation [64]. The SAR is computed with the following formula (Eq. 8):

(8) $$SAR=\frac{{Na}^{+}}{\sqrt{\frac{{Ca}^{2+}+{Mg}^{2+}}{2}}}$$

#### *2.3.6* Kelly's Ratio (KR)

Water that will be utilized for irrigation is categorized using Kelly's ratio. Values of KR (>1) and KR (<2) indicate an overabundance and shortfall of sodium, respectively, in fluids [65] Equation (9) was used to compute the KR. While waters with a high ratio are deemed undesirable, those with a low KR (<1) are suitable for irrigation [66].

(9) $$KR=\frac{{Na}^{+}}{\left({Ca}^{2+}+{Mg}^{2+}\right)}$$

#### *2.3.7* Permeability index (PI)

The amount of sodium, magnesium, calcium, and bicarbonate in the soil affects its permeability, which has a long-term impact effect on the quality of water used for agriculture. A standard for determining whether water is appropriate for irrigation was developed by Ref. [67] and based on the permeability index (PI), which was computed using Equation (10), where all ions were given in mg/L.

(10) $$PI=\frac{{Na}^{+}+\sqrt{{HCO}_{3}^{-}}}{{Ca}^{2+}+{Mg}^{2+}+{Na}^{+}}\times 100$$

### *2.4* Multivariate statistical analysis

Microsoft Excel 2011 was utilized in conjunction with GW Chart Software (USGS) to comprehend the hydrochemistry of groundwater. The software version Origin.9.65 (Origin Lab, USA) was utilized for the multivariate statistical analysis, principal component analysis (PCA), and hierarchical cluster analysis (HCA). Quantitative source apportionment of metals in this study was conducted with the EPA PMF5.0 model. The ArcGIS 10.1 program was used to create the GIS location map. Multivariate statistical analysis is commonly employed in identifying the origins of solutes within a groundwater system [68]. Using diverse multivariate methodologies, such as HCA and PCA, facilitates a greater understanding of water quality and enables the comparison of distinct water samples [69]. The groundwater quality data (TDS, pH, EC, DO, salinity, alkalinity, Na^+^, Cl^−^, Ca^2+^, Mg^2+^, SO_4_^2−^, K^+^, and HCO_3_^−^) were examined with a multivariate statistical method to test the reliability of the different processes that influence the mineralization of the groundwater aquifer system. The classification of water samples into various categories based on their hydrochemical properties was made possible by the combined use of PCA and HCA. These methods will identify groups and collections of variables having related characteristics.

## 3 Results and discussions

### 3.1 Physio-chemical properties of groundwater

Table 2, Table 3 provide an overview of the hydrochemical characteristics of collected samples. These tables also show that most of the parameters have significant standard deviations, indicating that various processes have influenced the chemical composition of the groundwater.

**Table 2:** Physiochemical parameters in shallow tubewell (STW) water.

Sample	pH	EC (mS/cm)	TDS (g/L)	DO (mg/L)	Salinity (%)	Alkalinity (mg/L)	Total Hardness (mg/L)
**STW-01**	7.70	5.34	2.81	7.36	2.90	355	2114.9
**STW-02**	7.15	8.56	3.65	5.24	3.20	175	447.2
**STW-03**	7.66	5.63	2.90	7.27	3.00	170	663.8
**STW-04**	7.25	6.75	2.81	7.19	2.80	210	1490.4
**STW-05**	7.60	4.86	1.25	7.25	1.50	255	960.8
**STW-06**	7.73	4.01	2.08	7.13	2.10	555	1700.6
**STW-07**	7.56	1.42	0.70	7.31	0.70	370	224.0
**STW-08**	8.46	6.26	3.31	7.28	3.40	255	211.8
**STW-09**	7.72	3.55	1.83	7.12	1.80	515	397.0
**STW-10**	7.94	3.07	1.57	6.84	1.60	805	223.7
**STW-11**	7.15	3.55	1.35	6.96	0.90	660	204.1
**STW-12**	7.63	2.33	1.02	7.32	0.70	555	207.4
**STW-13**	7.82	0.95	0.47	7.33	0.40	430	179.8
**STW-14**	7.20	0.97	0.48	7.23	0.50	215	184.8
**STW-15**	7.10	5.58	3.68	7.38	2.80	285	245.3
**STW-16**	7.50	2.48	0.80	7.29	0.90	275	123.3
**STW-17**	7.43	4.25	2.21	7.02	2.20	300	390.8
**STW-18**	7.70	9.01	4.88	7.17	5.00	260	1624.6
**STW-19**	7.80	3.23	1.65	7.26	1.70	285	246.3
**STW-20**	7.20	1.68	0.70	7.18	0.60	270	183.8
**STW-21**	7.86	2.20	1.29	1.54	1.70	195	290.2
**STW-22**	7.45	1.95	1.58	5.65	0.70	275	563.9
**STW-23**	7.23	1.16	0.5	1.18	0.60	640	258.8
**STW-24**	7.19	2.72	1.38	3.55	1.40	280	342.3
**Min**	7.10	0.95	0.50	1.18	0.40	170	123.3
**Max**	8.46	8.56	4.88	7.38	5.00	805	2114.9
**Mean**	7.54	3.81	1.87	6.41	1.80	357.9	561.7
**SD**	0.33	2.29	1.18	1.79	1.17	173	574.1
**WHO, 2011**	6.5–8	0.75	0.50	8.00	–	200	500

**Table 3:** Concentration of major ions and presence of heavy metals in STW.

Sample	**Ca**^**2+**^**(mg/L)**	**Mg**^**2+**^**(mg/L)**	**Na**^**+**^**(mg/L)**	**K**^**+**^**(mg/L)**	**Cl**^**−**^**(mg/L)**	**NO**_**3**_^**−**^**(mg/L)**	**HCO**_**3**_^**−**^**(mg/L)**	**SO**_**4**_^**2−**^**(mg/L)**	**Mn (mg/L)**	**Cu (mg/L)**	**Zn (mg/L)**	**Fe (mg/L)**	**As (μg/L)**
**STW-01**	248.5	355.6	876.0	1.5	18.2	1.53	433.1	0.0	1.396	0.012	0.011	0.30	3.75
**STW-02**	90.1	52.8	344.1	14.1	1043.3	1.24	213.5	70.3	0.014	0.019	0.014	0.41	0.58
**STW-03**	231.4	20.3	828.8	5.2	2.9	0.0	207.4	0.0	0.005	0.018	0.016	0.39	8.91
**STW-04**	287.2	183.9	428.2	2.5	5.9	0.0	256.2	0.0	0.243	0.013	0.065	0.25	0.03
**STW-05**	256.6	76.1	556.2	1.8	3.4	0.0	311.1	0.0	0.158	0.018	0.502	0.16	0.03
**STW-06**	133.3	380.1	154.0	8.1	1.7	0.0	677.1	0.0	0.005	0.021	0.025	0.13	0.47
**STW-07**	88.8	0.51	52.9	8.1	494.5	0.0	451.4	0.0	0.005	0.019	0.014	0.12	0.08
**STW-08**	41.8	25.6	573.0	24.1	1.7	0.0	311.1	0.0	0.001	0.024	0.059	0.15	0.29
**STW-09**	99.6	35.2	273.4	4.8	3.2	0.0	628.3	0.0	0.028	0.023	0.054	0.14	5.00
**STW-10**	56.8	19.5	313.8	4.5	1028.5	0.0	982.1	0.0	0.010	0.018	0.023	0.10	1.92
**STW-11**	61.2	12.2	344.1	3.0	1172.4	0.0	805.2	48.1	0.005	0.021	0.015	0.16	1.47
**STW-12**	63.3	11.7	166.9	2.3	642.8	0.90	677.1	4.9	0.006	0.022	0.017	0.11	2.73
**STW-13**	43.8	16.8	22.7	2.9	60.7	0.0	524.6	12.9	0.003	0.021	0.017	0.15	0.12
**STW-14**	50.8	13.8	28.3	2.0	54.5	0.80	262.3	0.0	0.007	0.020	0.027	0.14	0.82
**STW-15**	77.4	12.4	20.7	2.9	360.0	0.0	347.7	0.0	0.126	0.018	0.148	2.47	0.72
**STW-16**	41.8	4.5	8.6	2.8	38.0	0.0	335.5	0.0	0.014	0.019	0.024	0.35	0.87
**STW-17**	107.1	29.3	310.4	1.4	4.2	0.0	366.0	0.0	0.268	0.018	0.034	0.18	0.17
**STW-18**	269.2	226.6	566.3	1.2	4.7	0.0	317.2	0.0	0.066	0.015	0.031	0.16	0.10
**STW-19**	67.8	18.3	194.1	3.1	1419.2	0.0	347.7	0.0	0.021	0.017	0.021	0.18	9.34
**STW-20**	48.1	15.2	51.5	2.7	929.8	0.0	329.4	0.0	0.020	0.012	0.016	0.19	4.30
**STW-21**	57.3	34.9	424.9	9.1	2.3	0.0	337.9	0.0	0.018	0.014	0.011	0.14	0.66
**STW-22**	97.6	76.2	589.8	11.1	8.8	0.0	335.5	0.0	0.018	0.002	0.013	0.09	2.83
**STW-23**	43.9	35.5	22.9	7.9	180.9	0.0	780.8	0.0	0.009	0.020	0.011	0.13	0.20
**STW-24**	81.2	33.2	169.8	9.0	729.0	0.0	341.6	0.0	0.028	0.014	0.012	0.14	0.36
**Min**	41.8	0.51	8.6	1.2	1.7	0.0	207.4	0.0	0.001	0.002	0.014	0.11	0.03
**Max**	269.2	380.1	876.0	24.1	141.9	1.53	982.1	70.3	1.390	0.028	0.500	2.47	9.34
**Mean**	110.2	70.4	305.1	5.7	342.1	0.20	440.8	5.7	0.103	0.017	0.048	0.28	1.90
**SD**	81.6	106.3	257.3	5.2	463.9	0.40	207.9	17.0	0.279	0.006	0.090	0.46	2.59
**WHO**	75	30	200	30	250	45	200	200	0.500	0.050	–	0.1	0.01

Its average pH value was found to be 7.54, and it ranged from 7.1 to 8.46. Consequently, it was inferred that the water was neutral to slightly alkaline [70]. The pH recommendation for irrigation water was 6.5–8.0 [71]. The mean Dissolved Oxygen (DO) concentration was 4.61 mg/L, whereas the minimum concentration of DO was 1.18 mg/L, and the maximum concentration of DO was 7.38 mg/L. All water samples except STW-02, STW-21, STW-22, STW-23, and STW-24 DO values were higher than the Department of Environment (DoE)-set requirement of 6.00 mg/L or greater for Bangladesh drinking water [72].

Total dissolved solids (TDS) were 0.5–4.88 g/L, with an average value of 1.87 g/L. Increased salinity, sodium, and chloride ion concentrations might be responsible for the excessive TDS level. According to the TDS classification, 25% of the wells were freshwater, and the rest of the tubewell were brackish water (TDS>1) [73]. The International Organization for Standardization rates drinking water's palatability to its TDS level as follows: Excellent is less than 0.3 g/L, followed by good, between 0.6 and 0.9 g/L, fair, between 0.9 and 1.2 g/L, poor, and unacceptable at more than 1.2 g/L [74]. As a result, all TDS levels were unacceptable except STW-07, STW-12, STW-13 STW-14, STW-16, and STW-23.

Drinking and irrigation water have been categorized using electrical conductivity (EC) as a standard [75]. The range of the EC is 0.95–8.56 mS/cm, with means of 3.81 mS/cm. The [71] guidelines state that the acceptable limit of EC is 0.75 mS/cm. All of the samples were above the average EC value.

**Fig. 2:**
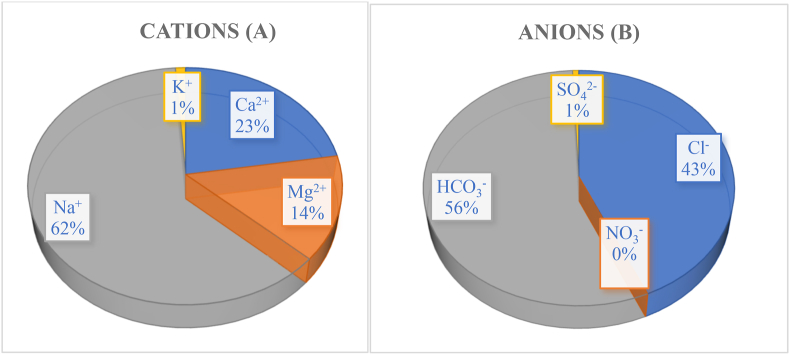
Pie diagram to represent the contribution (%) of different cations (A) and anions (B) of the study area.

The salinity, TDS, and EC have a strong correlation with one another [76]. This study showed that the TDS and EC values were similarly excessive when the salinity of tubewell water was 0.5% or greater [77]. The concentration of Na^+^, Ca^2+^, Mg^2+^ and K^+^ corresponded to mean value of 62%, 23%, 14%, and 1%, respectively (Fig. 2A). The concentration of Cl^−^, HCO_3_^−^, SO_4_^2−^, CO_3_^2−^, and NO_3_^−^ contributed an average of 43%, 56%, 1%, and 0% (Fig. 2B) of the total anions. The water of the studied region was dominated by Na^+^, Ca^2+^, Mg^2+^, Cl^−^, and HCO_3_^−^ (Fig. 2).

Due to excessive water removal, decreased river discharge rates, and rock weathering, saline water originating from the Bay of Bengal may have invaded the land. This may explain the high Na^+^, Ca^2+^, Mg^2+^, and Cl^−^ concentrations. The HCO_3_^−^ and CO_3_^2−^ concentrations exhibited significant variability, but the anion contribution (%) indicated that the first one predominates in the studied area. This can be caused by both the dissolution of the carbonic acids and the weathering of carbonate [59,60]. Most of the studied region was heavily irrigated [71]. The high concentration of nitrate in some areas might be caused by the suggested agricultural fertilizers [78]. The average Mn concentration was 0.10 mg/L, exceeding the (<0.05 mg/L) WHO recommendation for drinking water. These increased levels of Fe and Mn may cause low pH levels in the studied area.

### 3.2 Status of heavy metals

This study found a strong correlation between arsenic contamination of tubewell water and the outcomes [22]. All samples had an excessive level of arsenic present. Arsenic was naturally found in more than 60% of Bangladesh's groundwater, with concentrations frequently surpassing 10 μg/L [79]. Long-term use of iron-rich water can cause a severe condition known as hemosiderosis [80]. The presence of high levels of arsenic in the groundwater can likely be accused for other illnesses, including liver and kidney damage, skin cancer, and breast cancer [81].

Out of all samples, fifteen (15) had water with higher iron (Fe) than the recommended limit for Bangladesh guided by the WHO reference level, which is 0.1 mg/L, but the permitted maximum limit in Bangladesh is 0.3–1.0 mg/L. Approximately 85.75 % of the tubewells with noteworthy iron contamination whose depth was under 90 feet [82]. According to Table 3, there is no lead contamination in the water from tube wells in the research region.

Pearson's correlation matrix was used to determine the relationships between the various water quality metrics. Table 4 shows statistically significant positive associations among EC and TDS (r = 0.93). TDS and EC demonstrated a highly substantial correlation with the ions Mg^2+^, Ca^2+^, Na^+^, K^+^, NO_3_^-^, SO_4_^2−,^ and Cl^−^. Water in the studied area is highly salinized, and precipitation from agricultural chemicals and seawater intrusion into the groundwater aquifers may have contributed significantly to the result. Moreover, total hardness (TH) demonstrated strong positive correlations with EC, TDS, Mg^2+^, Ca^2+^, Na^+^, K^+^, Fe, and Cl^−^, whereas it had substantial negative correlations with pH and HCO_3_^−^ anion. Principal Component Analysis (PCA) further confirmed this.

**Table 4:** Pearson's correlation Matrix of physiochemical parameter of analyzed water (STW) (n = 24).

	pH	EC	TDS	DO	Salinity	Alkalinity	Ca^2+^	Mg^2+^	Na^+^	K^+^	Cl^−^	NO_3_^−^	HCO_3_^−^	SO_4_^2-^	TH
pH	1														
**EC**	0.07	1													
**TDS**	0.12	0.93	1												
**DO**	0.17	0.24	0.21	1											
**Salinity**	0.25	0.93	0.96	0.17	1										
**Alkalinity**	0.10	−0.34	−0.33	−0.03	−0.34	1									
**Ca**^**2+**^	0.01	0.63	0.54	0.26	0.60	−0.31	1								
**Mg**^**2+**^	0.12	0.40	0.41	0.13	0.46	0.02	0.62	1							
**Na**^**+**^	0.36	0.56	0.54	0.09	0.58	−0.26	0.67	0.40	1						
**K**^**+**^	0.37	0.14	0.18	−0.31	0.17	−0.13	−0.30	−0.10	0.15	1					
**Cl**^**−**^	−0.23	−0.08	−0.13	0.01	−0.20	0.28	−0.39	−0.34	−0.28	−0.02	1				
**NO**_**3**_^**−**^	−0.17	0.20	0.13	0.07	0.13	−0.24	0.09	0.29	0.17	−0.03	−0.02	1			
**HCO**_**3**_^**−**^	0.12	−0.36	−0.34	−0.09	−0.35	0.99	−0.32	0.01	−0.25	−0.12	0.27	−0.28	1		
**SO**_**4**_^**2−**^	−0.24	0.30	0.21	−0.03	0.11	−0.07	−0.14	−0.12	0.08	0.20	0.34	0.38	−0.04	1	
**TH**	0.09	0.54	0.51	0.19	0.56	−0.11	0.83	0.95	0.56	−0.19	−0.39	0.27	−0.12	−0.14	1

### 3.3 Principal component analysis (PCA)

**Fig. 3:**
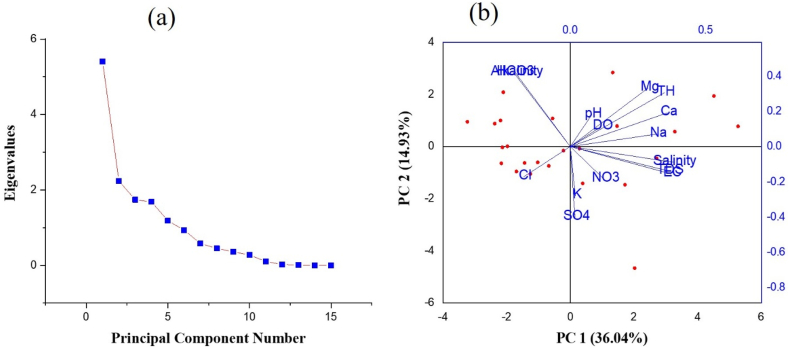
Principal component analysis (PCA) scree plot (a) and biplot (b) based on the correlation matrix.

PCA is a technique that complements traditional hydrogeochemical research methodologies by showing an association between various water quality metrics and allowing for quick visualization [83]. PCA identified five variables with Eigenvalues greater than one on the combined datasets, and they may account for about 81.72% of the data's variability (PC1 variance: 36.04%; PC2 variance: 14.93%) (Table 5) (Fig. 3 a, b).

**Table 5:** Eigenvalues and Extracted Eigenvectors of the Correlation Matrix for principal component analysis (PCA).

Component	Eigenvalue	Variance%	Cumulative%	PC1	PC2
pH	5.40644	36.04	36.04	0.08	0.18
**EC**	2.23910	14.93	50.97	0.37	−0.15
**TDS**	1.74202	11.61	62.58	0.36	−0.14
**DO**	1.68397	11.23	73.81	0.11	0.11
**salinity**	1.18672	7.91	81.72	0.38	−0.09
**alkalinity**	0.93262	6.22	87.94	−0.20	0.42
**Ca**^**2+**^	0.58016	3.87	91.81	0.36	0.19
**Mg**^**2+**^	0.45061	3.00	94.81	0.29	0.33
**Na**^**+**^	0.36301	2.42	97.23	0.32	0.07
**K**^**+**^	0.27710	1.85	99.08	0.02	−0.28
**Cl**^**−**^	0.10389	0.69	99.77	−0.17	−0.17
**NO**_**3**_^**−**^	0.02445	0.16	99.93	0.12	−0.18
**HCO**_**3**_^**−**^	0.00754	0.05	99.98	−0.21	0.42
**SO**_**4**_^**2-**^	0.00161	0.01	99.99	0.02	−0.40
**TH**	0.00077	0.01	100.00	0.35	0.31

When two variables are close to each other and far from the center of the biplot analysis of PCA (Fig. 3b), it can be inferred that these variables are significantly and positively correlated (r = 1). Na^+^, Mg^2+^, and Ca^2+^ are PC1 coefficients that may be related to the overall hardness of the groundwater. The groundwater salinity, which is directly related to EC, TDS, and Cl^−^, may be a factor in PC 2 (Fig. 3).

### 3.4 Hierarchical cluster analysis (HCA)

**Fig. 4:**
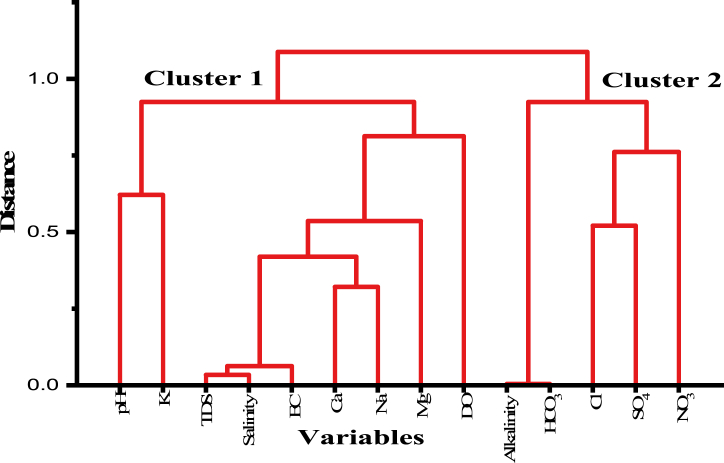
Dendrogram for all the physio-chemical parameters of the water samples.

The correlation index and dendrogram can be used to interpret how various physio-chemical parameters relate. The statistical method known as hierarchical cluster analysis (HCA) is employed to partition a given dataset into distinct groups by considering their degree of similarity. The dendrogram provides a visual representation of the data set, enabling the analysis of the standard deviations within the variations of the data set [84]. Based on the commonalities in the samples taken from various places around the studied area, the various water quality parameters or sampling locations can be categorized [[85], [86], [87]]. The closest pair's similarity is combined into a cluster in the current dendrogram (Fig. 4) using a single linkage (group). The horizontal axis displays the proximity of the data sets with close correlations, while the vertical axis shows the distance between the data sets based on the values of the concentrations of each data parameter [88].

The dendrogram depicts the relationship between the water sample concentrations for several factors in the current studied region of Kaligonj. The graphic suggests that there is a correlation between the variations in the values of K^+^ and pH, indicating compatibility. However, significant variations are observed in TDS, salinity, EC, Ca^2+^, Na^+^, Mg^2+^, DO, alkalinity, HCO_3_^−^, Cl^−^, SO_4_^2−^, NO_3_^−^. Varying seawater intrusions at various locations might cause this variance because each place is almost equally close to the coast [89].

### 3.5 Quantitative source apportionments

As the final computation in this study was adjusted to have a reduced error (%), the value of 0.2 for C was chosen, and the Q true/Q exp ratio was 1.18. Additionally, 5% uncertainty was used to reduce technique mistakes in preparing the filter papers, gravimetric mass measurement, and calibration curves. The source apportionment approach is commonly used in the literature to calculate the uncertainty of the variables by using PMF 5.0 equations above [[90], [91], [92], [93], [94]].

**Fig. 5:**
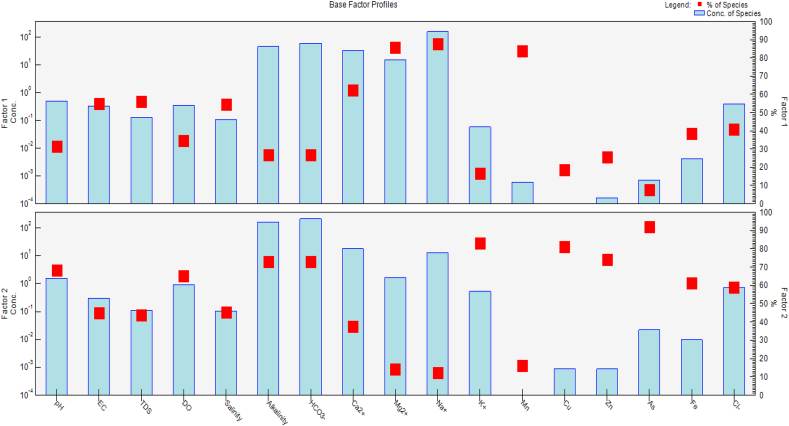
PMF factor profiles for identifying sources by bars (left y-axis) and the percentage contributions (right y-axis).

Finding the ideal number of components (p) is crucial when using the PMF model, and the p-value was established by looking at Q values for PMF solutions. The number of components was used to be determined by Pearson's correlation analysis [95] to minimize the level of uncertainty of the model outcomes. The metals may have a similar origin and transport path, given the substantial correlations between two or more elements [96]. These findings implied two possible origins in the investigated area (Fig. 5). Two components were evaluated using the PMF (Fig. 5), and their profiles and contributions to the studied metals are shown in Table 6. Factor 1 was heavily characterized by EC, TDS, salinity Ca^2+^, Mg^2+^, Na^+,^ and Mn were responsible for 54.8%, 56.2%, 54.5%, 62.5%, 85.8%, 87.7%, and 84%, respectively. This may come from seawater intrusion, indicating the presence of significant saline water in the sampling area. The second factor contributed 68.6%, 65.2%, 73.1%, 83.1%, 81.3%,74.3%,61.5%, 92.3%, and 58.9% of pH, DO, alkalinity, K^+^, Cu, Zn, Fe, As, and Cl^−^ respectively. This distribution inferred that factor 2 might be mostly sourced from anthropogenic activities in the studied area.

**Table 6:** Distribution of elements among the different factors inferred from the PMF model.

Parameters	Factor 1	Factor 2
pH	31.4	68.6
**EC**	54.8	45.2
**TDS**	56.2	43.8
**DO**	34.8	65.2
**Salinity**	54.5	45.5
**Alkalinity**	26.9	73.1
**HCO**_**3**_^**−**^	27.0	73.0
**Ca**^**2+**^	62.5	37.5
**Mg**^**2+**^	85.8	14.2
**Na**^**+**^	87.7	12.3
**K**^**+**^	16.9	83.1
**Mn**	84.0	16.0
**Cu**	18.7	81.3
**Zn**	25.7	74.3
**As**	7.7	92.3
**Fe**	38.5	61.5
**Cl**^**−**^	41.1	58.9

### 3.6 Classification of ground water and chloroalkaline index

**Fig. 6:**
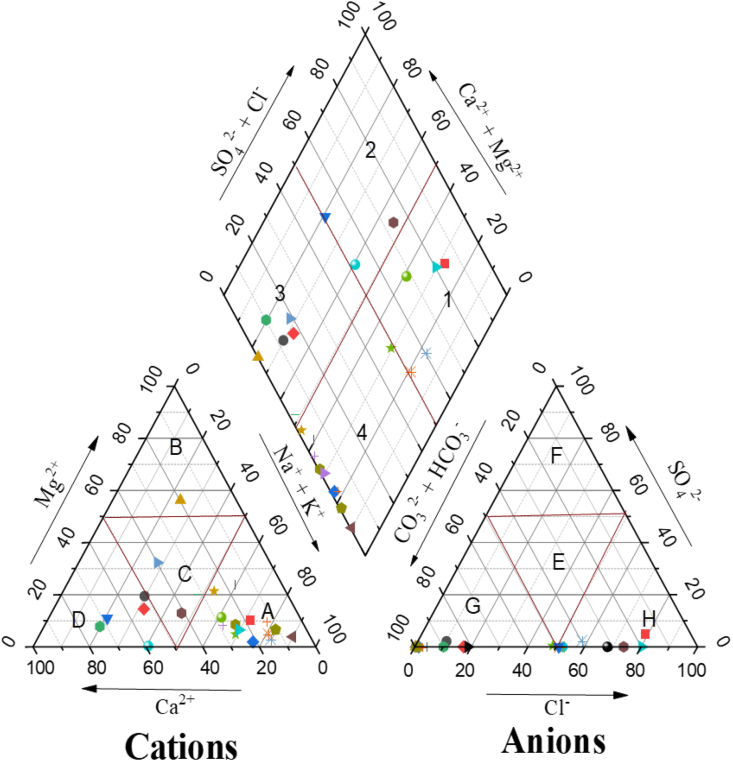
Piper diagram of the samples in the studied region [(1) SO_4_^2−^ type, (2) Ca^2+^-Mg^2+^-SO_4_^2-^-Cl^-^ type, (3) Ca^2+^-Mg^2+^- HCO_3_^−^ type, (4) Na^+^- HCO_3_^−^ type, (A) Na^+^- K^+^ type, (B) Mg^2+^ type, (C) No-dominance, (D) Ca^2+^ type, (E) No-dominance, (F) SO_4_^2−^ type, (G) Cl^−^ type, and (H) HCO_3_^−^ type].

To comprehend the various types of groundwater bodies with varying chemical characteristics and compositions, groundwater classifications are used [97]. The hydrochemical characteristics of groundwater vary according to the lithology, regional water flow patterns, and resident time [98]. Based on its chemical makeup, all water can be categorized into three (3) classes: Cl^−^, SO_4_^2−^, and HCO_3_^−^ types [99]. The type of water can be determined using the Piper diagram (Fig. 6). The central portion of the diagram has a diamond form, while the lower section features two trilinear diagrams.

The trilinear diagram shows each sample's cation (left diagram) and anion (left diagram) relative concentrations. For a Piper diagram, cations are divided into three (3) primary categories: (Na^+^+ K^+^), Ca^2+^, and Mg^2+^. Similarly, (HCO_3_^−^ + CO_3_^2−^), Cl^−^, and SO_4_^2−^ are the three (3) main groups for anions. In the trilinear diagram, each water sample is graphically represented as a point, while the classification of water samples is decided by the symbolic area depicted in the Piper diagram. Fig. 6 illustrates the significant degree of main ion chemistry variability. Around 63% of the samples were of the Na^+^-K^+^-Cl^-^-SO_4_^2-^ type, and about 72% were sodium-potassium and alkali types in the studied area. 98% of samples are covered in chloride and bicarbonate. (1) SO_4_^2−^ type, (2) Ca^2+^-Mg^2+^-SO_4_^2-^-Cl^-^ type, (3) Ca^2+^-Mg^2+^- HCO_3_^−^ type, (4) Na^+^- HCO_3_^−^ type, (A) Na^+^- K^+^ type, (B) Mg^2+^ type, (C) No-dominance, (D) Ca^2+^ type, (E) No-dominance, (F) SO_4_^2−^ type, (G) Cl^−^ type, and (H) HCO_3_^−^ type (Fig. 6).

Positive CAIs show that Mg^2+^ and Ca^2+^ from the rocks and Na^+^ and K^+^ from the water have directly exchanged ions. Moreover, CAIs are indirect and harmful when Mg^2+^ and Ca^2+^ from the water exchange ions with Na^+^ and K^+^ from the rocks. The CAIs (1, 2) were calculated in this work, and Table-7 displays the results. The findings demonstrated that 45.83% of the aquifer samples in the research sites had negative CAIs, while 54.16% had positive CAIs.

**Table 7:** Concentrations of water quality index for irrigation of the studied area.

Sample	% Na^+^	RSC (mg/L)	SAR (mg/L)	KR (mg/L)	PI %	CAI1 (mg/L)	CAI2 (mg/L)
**STW-01**	59.2	−171.0	50.4	1.44	60	−47.2	−2.00
**STW-02**	71.5	70.6	30.7	2.40	73	0.70	3.20
**STW-03**	76.8	−44.3	73.9	3.30	116	−285.6	−4.00
**STW-04**	47.8	−214.9	27.9	0.90	49	−72.2	−1.70
**STW-05**	62.7	−21.5	43.1	1.70	64	−163.7	−1.80
**STW-06**	24.0	163.8	9.6	0.30	152	−93.8	−0.20
**STW-07**	40.9	362.1	7.9	0.60	52	0.90	1.00
**STW-08**	89.9	243.8	98.6	8.50	92	−356.5	−1.90
**STW-09**	67.4	493.5	33.3	2.00	75	−86.0	−0.40
**STW-10**	80.7	905.9	35.9	4.10	88	0.70	0.70
**STW-11**	82.6	731.8	56.8	4.70	89	0.70	1.00
**STW-12**	69.3	602.1	27.3	2.20	88	0.70	0.70
**STW-13**	29.7	464.1	4.1	0.40	54	0.60	0.10
**STW-14**	31.9	197.7	3.5	0.40	47	0.40	0.10
**STW-15**	20.8	258.0	3.1	0.20	51	0.91	1.00
**STW-16**	19.8	289.2	1.8	0.20	48	0.70	0.10
**STW-17**	69.6	229.6	37.6	2.30	73	73.2	0.80
**STW-18**	53.4	−178.6	25.4	1.10	71	120.2	1.80
**STW-19**	69.6	261.6	20.9	2.30	75	0.80	3.50
**STW-20**	46.1	266.2	6.5	0.80	60	0.90	2.70
**STW-21**	82.5	245.8	44.3	4.60	71	−188.5	−1.30
**STW-22**	77.6	161.8	63.3	3.40	49	−67.3	−1.80
**STW-23**	27.9	701.3	3.6	0.30	79	0.80	0.20
**STW-24**	61.0	227.2	22.5	1.50	76	0.80	1.60
**Min**	19.76	−214.9	1.8	0.20	47	−356.5	−4.00
**Max**	89.86	905.9	98.6	8.50	152	120.2	3.50
**Average**	56.77	260.2	30.5	2.10	73	−48.2	0.10
**SD**	21.49	281.0	24.6	1.90	23.61	104.7	1.80

### 3.7 Major ion chemistry

**Fig. 7:**
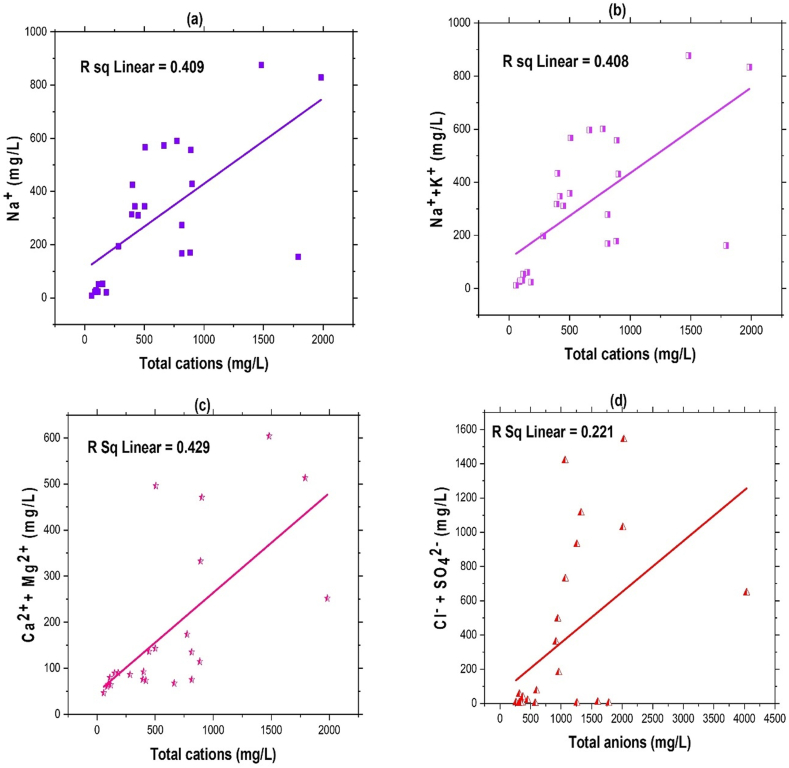

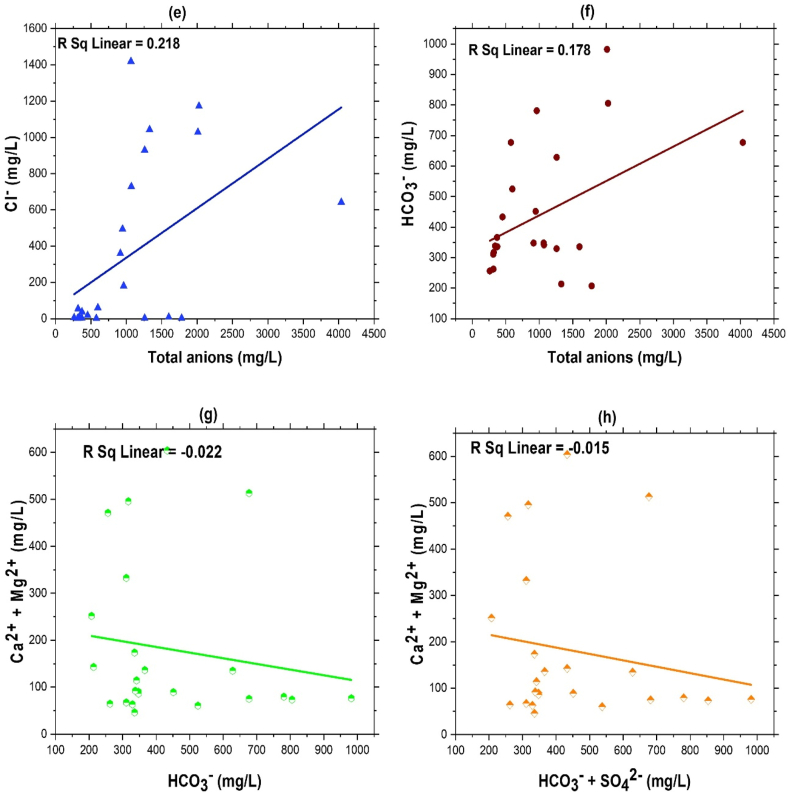
Ion scatter diagram (a–h) showing relationships among major ions in the groundwater (STW) where (a) Na^+^ vs total cations (b) Na^+^ + K^+^ vs total cations (c) Ca^2+^ + Mg^2+^ vs total cations (d) Cl^−^ + SO_4_^2−^ vs total anions (e) Cl^−^ vs total anions (f) HCO_3_^−^ vs total anions (g) Ca^2+^ + Mg^2+^ vs HCO_3_^−^and (h) Ca^2+^ + Mg^2+^ vs HCO_3_^−^ + SO_4_^2−^.

The equiline plot (Fig. 7) for the different ions displays the affinities and properties of the ions. Most values on the equiline in Na^+^ vs total cation plot indicate that the total cations balance the alkali ions. Also, the ratio of Na^+^ to total cations (Fig. 7a) was 0.409, and the ratio of Ca^2+^ + Mg^2+^ to total cations (Fig. 7c) was also discovered to be 0.429, showing that total cations were the primary source of balance for the majority of the ions. Na^+^ was the most prevalent alkali, whereas K^+^ content appeared low. The ratios of Na^+^ + K^+^ and Cl^−^ + SO_4_^2−^ to total cations and anions of 0.408 and 0.221, respectively (Fig. 7b,d), demonstrate the supremacy of alkalis over alkaline earth metal ions. The ratio of Cl^−^ to total anions (Fig. 7e) was 0.218, and that of HCO_3_^−^ to total anions (Fig. 7f) was 0.178, demonstrating the supremacy of HCO_3_^−^ over the alkaline metal ions. Ca^2+^ + Mg^2+^ had a ratio of −0.0.022 to HCO_3_^−^ and −0.015 (Fig. 7g) to HCO_3_^−^ + SO_4_^2−^ (Fig. 7h), respectively. The generation of HCO_3_^−^ ions in groundwater water is frequently attributed to the chemical reaction between carbonate ions and water molecules. This process results in the release of hydroxyl ions into the water, leading to an increase in pH and the manifestation of alkalinity.

**Fig. 8:**
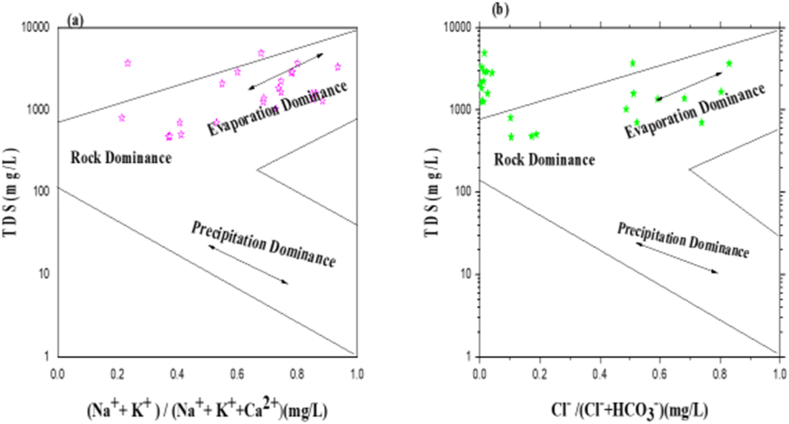
The Gibb's Ratios of (a) TDS versus cations and (b) TDS versus anions.

The explanation of the chemical reactions process leading to alterations in the composition of groundwater and sources is illustrated by using Gibb's diagram [100]. Fig. 8a and b shows two charts representing TDS versus (Na^+^ + K^+^)/(Na^+^ + K^+^ + Ca^2+^) and TDS vs Cl^−^/(Cl^−^ + HCO_3_^−^), respectively. Fig. 8a and b demonstrate that roughly 75% of the water samples fell in the rock and evaporation dominance regions. It is intriguing to observe that the occurrence of weathering response in the studied area is clearly described by both the cation and anion plots.

### 3.8 Status of drinking and irrigation water suitability

**Fig. 9:**
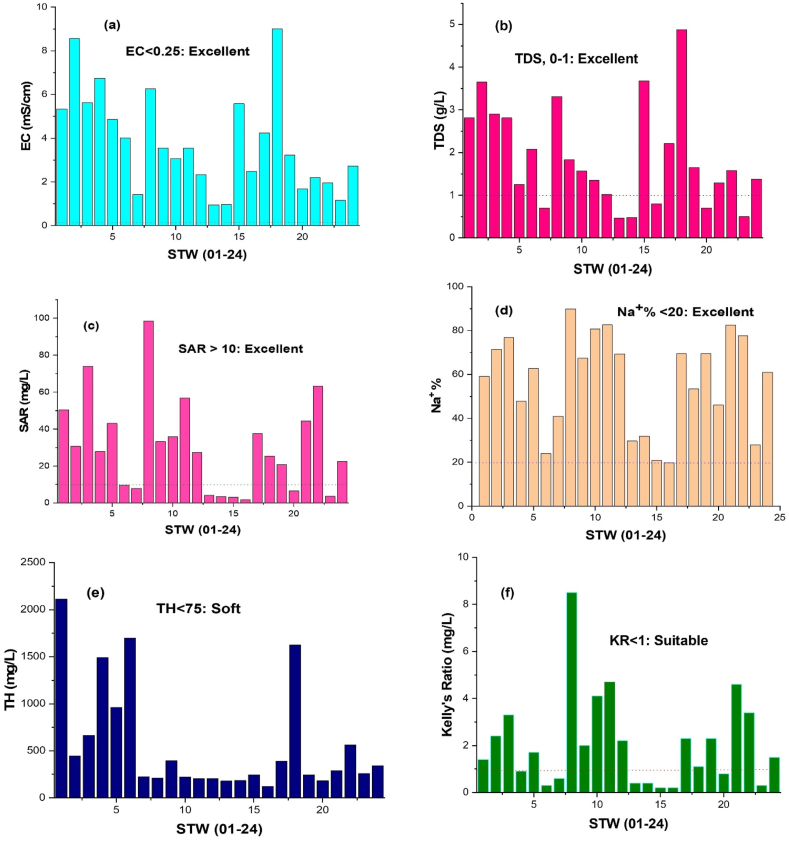
EC, TDS, SAR, % Na^+^, TH, KR versus concentration of Groundwater in different locations.

In Table 8, the samples' classification is displayed. According to the classification made by Ref. [101], 4% of groundwater falls into the excellent class, and 54% falls into the questionable range (Table 8) (Fig. 9d). As samples from the category of acceptable and permitted for irrigation purposes decrease, the effect of dilution becomes apparent. In general, greater Na^+^% was seen, which suggests that lithological units are weathering and exchanging ions.

**Table 8:** Classification of water sample according to different types of parameters.

Parameters	Reference	Range	Classification	N. of samples within std. range
**EC**	Wilcox (1955)	<0.25 mS/cm 0.25–0.75 mS/cm 0.75–2.25 mS/cm 2.25–5 mS/cm >5 mS/cm	Excellent Good Permissible Doubtful Unsuitable	0 0 7 10 7
**SAR**	Richards (1968)	<10 mg/L 10–18 mg/L 18–26 mg/L >26 mg/L	Excellent Good Doubtful Unsuitable	8 0 3 13
**TDS**	Freeze and Cherry (1979)	0–1 g/L	Freshwater	6
**TH**	Sawyer and McCarty (1967)	0–75 mg/L 75–150 mg/L 150–300 mg/L	Soft Moderately hard Hard	0 1 6
**RSC**	Eaton (1950)	<1.25 meq/L	Suitable	5
**Kelly's ratio**	Kelly (1940)	<1	Suitable	9
**% Na**^**+**^	Wilcox (1955)	<20 20–40 40–60 60–80	Excellent Good Permissible Doubtful	1 5 5 12

RSC averaged 260.2 mg/L and varied from −214.9 to 905.9 mg/L (Table 7). The findings showed that 80% of the samples weren't appropriate for irrigation (Table 8). Table 8 displays the groundwater suitability classification based on the SAR value. According to the results, 65% of the groundwater samples fell into the unsuitable group, and the remaining 12–27% of samples were found to be in the suitable–doubtful range (Fig. 9c).

KR varied from 0.2 to 8.5 mg/L (Table 7), and (Fig. 9f) 37% of the total samples were appropriate, while the remaining samples were in the inappropriate range (Table 8). The KR results showed that weathering of Feldspars from the studied area's litho units could produce a larger percentage of Na^+^ [102]. Both irrigation and drinking from the groundwater were ineligible uses.

**Fig. 10:**
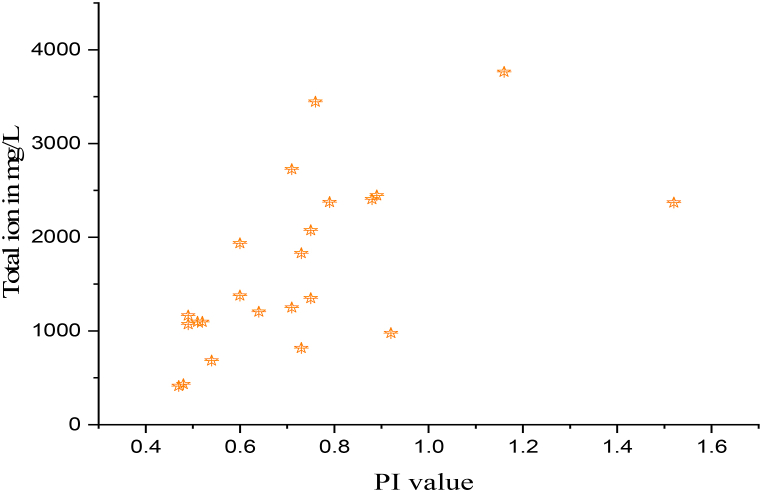
Total ion concentrations versus Permeability index (PI).

The Permeability index (PI) categorizes water into three levels such as class I, class II, and class III. Class I and Class II water is characterized as suitable for agriculture when it has a maximum PI of 75% or above, whereas Class III water is unsuitable when it has a maximum permeability of 25%. The PI used in this investigation ranged from 47 to 152%, averaging 73%. Groundwater was more suited for irrigation because the maximum PI values were between Class II and Class I (Fig. 10).

The acceptable limit of TDS is 0.5 g/L, while the highest allowable limit is 1.5 g/L (WHO 1996, 2011). TDS levels in the research region ranged from 0.5 to 4.88 g/L, showing that 74% of water samples were above the recommended limit of 0.5 g/L (Table 2) (Fig. 9b). The groundwater was categorized as per [103] total hardness (TH) scale. The 0.08–0.1 g CaCO_3_/L range was the most preferred limit for TH [73]. The average TH was 561.7, ranging from 123.3 to 2114.9 mg/L, falling into the very hard water categories (Table 8) (Fig. 9e). Hence, it is established that 85% of the samples were unfit for household use. Moreover, EC demonstrated an extremely high salinity level, with just 5% of samples falling into the medium salinity range and none falling within the low salinity range [101] (Table 8) (Fig. 9a). The utilization of irrigation water sourced from tubewells, which contains notable chemical elements derived from both natural sources and human activities, has been observed to have detrimental effects on crop yield and soil fertility [104,105]. The utilization of water for irrigation facilitates the transportation of salts to the zone surrounding the roots [106,107]. For the irrigation water suitability test, variables including the SAR, % Na, RSC, and PI are being taken into consideration [108]. Since salt water conducts electricity well, the EC of water is a measure of its level of electrical conductance. The findings of the chemical examination revealed that the research area's salinity level was categorized (Table 8) according to the US salinity laboratory [101]. No sample fell into the excellent category.

### 3.9 Spatial distribution maps for selected parameters

**Fig. 11:**
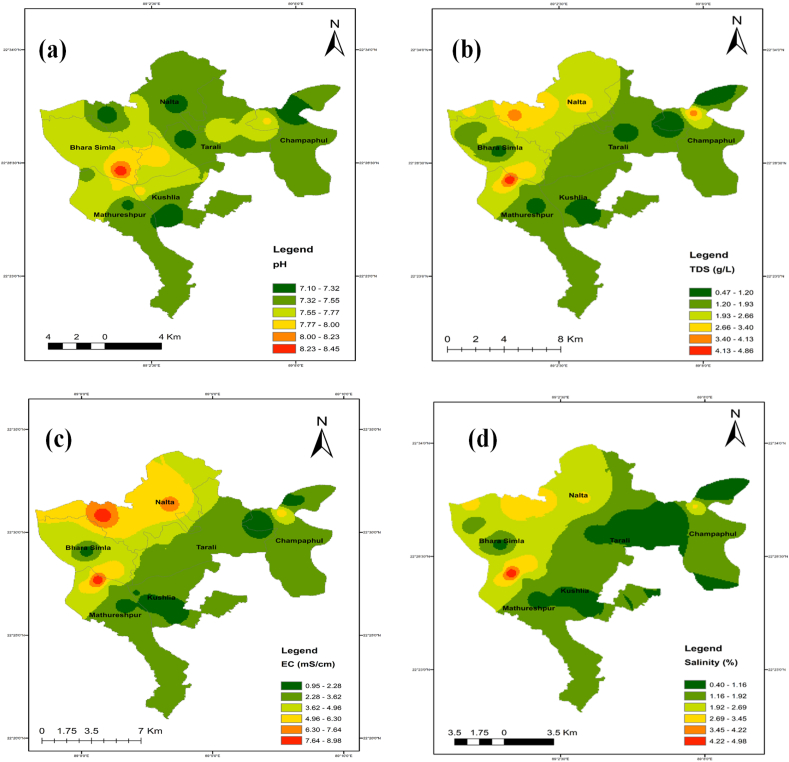

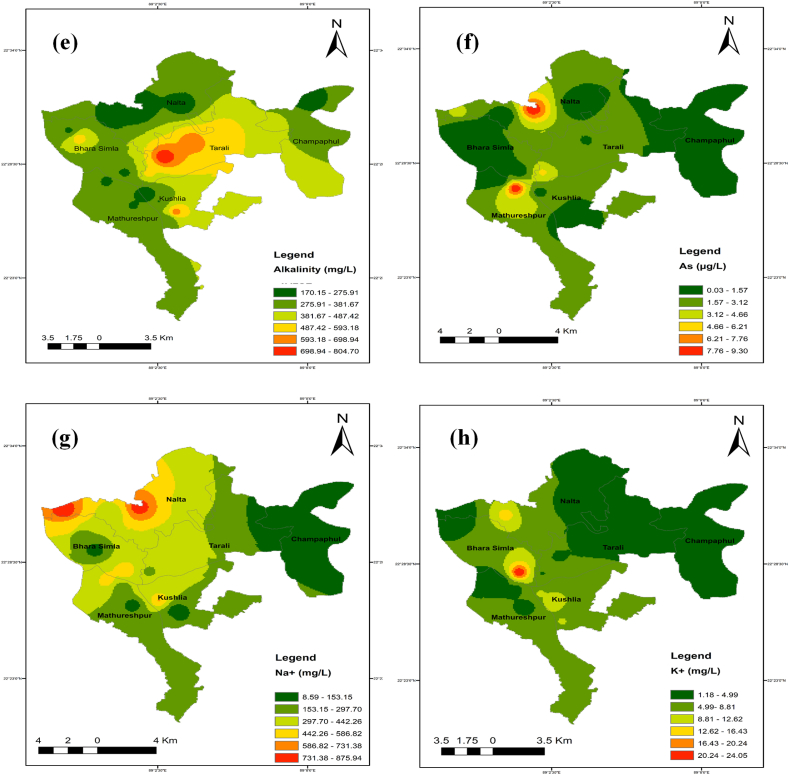

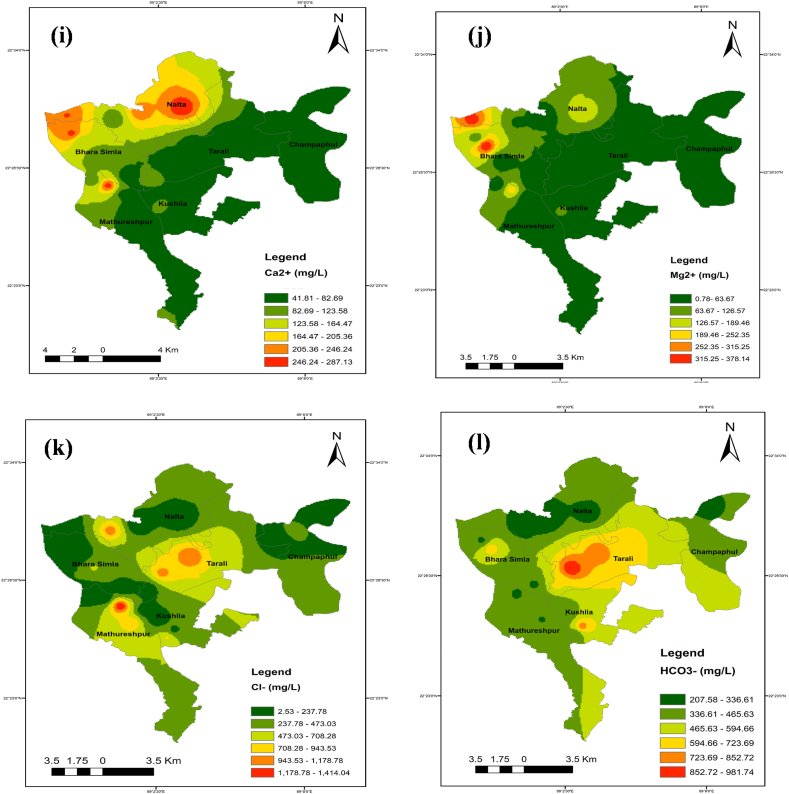

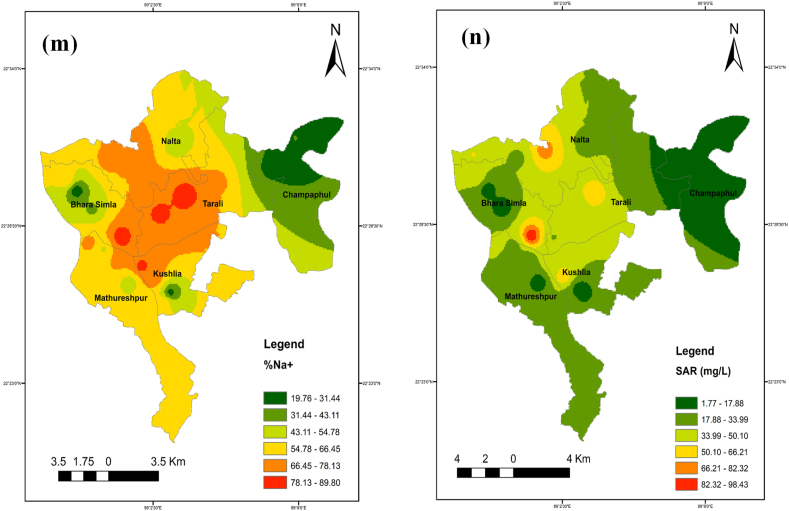
Spatial distribution maps (a–n) for selected parameters while (a) pH, (b) TDS, (c) EC, (d) Salinity, (e) Alkalinity, (f) As, (g) Na^+^, (h) K^+^, (i) Ca^2+^, (j) Mg^2+^, (k) Cl^−^, (l) HCO_3_^−^, (m) % Na^+^ and (n) SAR.

The identical spatial distribution maps of the TDS, chloride, sodium, magnesium, and calcium concentrations show where salinity first appeared. Spatial distribution maps were created with ArcGIS software using the IDW technique to analyze the spatial extent of the water quality metrics throughout the full studied region (Fig. 11 a-n).

In the northwest of the studied region, it was found that the values for EC, TDS, salinity, calcium, magnesium, sodium, manganese, and arsenic concentrations were greater. Seawater intrusion can easily influence shallow groundwater, whereas deep groundwater is more contamination-resistant. Yet, anthropogenic activities and river flow may have also had a role in this. The middle region of the research area had greater SAR and %Na^+^ values, indicating more occurrences. The geographic distribution of the criteria as mentioned earlier indicates that the area is impacted by very saline water originating from the ocean.

### 3.10 Implications, conservation and adoption

The salinity issue in coastal regions of Bangladesh has been on the rise [109]. The coastal rural communities are facing significant challenges in dealing with the escalating levels of soil and water salinity. Studies on the effects of water salinity on health have primarily focused on the southwestern regions of Bangladesh. Villagers residing in the study areas have been enduring the persistent issue of highwater salinity for an extended period of time, resulting in numerous health and livelihood challenges. The salinity issues in coastal areas are worsening due to unfavorable climatic conditions such as rising sea levels, frequent cyclones, and prolonged drought. This poses a significant risk to household water supplies [[110], [111], [112]]. The origin and distribution of the physicochemical characteristics of water in the study area are important insights provided by this study. The results of this study can be an asset for groundwater management projects in Bangladesh's coastal areas. Our approach can serve as a useful model for comparable situations, particularly in developing and tropical nations.

Rainwater harvesting is an alternative source of drinking water in the salinity prone area [110]. In addition, they proposed conducting further research to enhance the administration of fresh surface water and groundwater resources in regions affected by salt, as well as investigating rainwater harvesting as a viable and sustainable approach. Rainwater harvesting is an inexpensive technology [44] that can be encouraged in areas affected by salinity and areas with a shortage of fresh water. Harvested rainwater can be utilized for both domestic and agricultural applications. The Bangladesh government has actively promoted the conservation of rainwater. Rainwater harvesting techniques should be implemented to mitigate the drinking water crisis in the coastal area [113]. Establishing and upkeeping ponds could serve as an alternative approach for the preservation and exploitation of surface water. The ponds would serve various functions, such as fishing, bathing, and even for agricultural endeavors. These measures would enhance the socio-economic conditions and ensure greater food security for the population. The pertinent governmental and non-governmental organizations could promote and offer incentives to rural individuals for the establishment of ponds and rainwater-harvesting devices [43,114]. The primary difficulty in implementing pond sand filters in the study area lies in locating ponds that meet the criteria of being perennial, devoid of fish farming, and safeguarded against activities such as bathing, washing clothes, and watering cattle. The water utilized in the pond sand filtration process is sourced from ponds that are susceptible to contamination and serve as conduits for waterborne illnesses. Hence, it is crucial to establish and enforce measures aimed at safeguarding the ponds from any potential sources of contamination. In addition to rainwater harvesting and pond sand filters, the acquisition of potable water is deemed safe for consumption. This research has the potential to make significant contributions to the assessment of water quality for drinking purposes. This also offers an assessment of the suitability of drinking water, which can be valuable for evaluating the quality of water that is safe to drink, taking into account the potential risks to human health. It also examines the presence of contaminants in the groundwater of the study area and their origin. This study can serve as a suitable benchmark for local and regional water stakeholders, and this index can be implemented in other regions worldwide. If these events occur, there is a possibility that the individuals residing in the study area will experience some degree of relief from salinity issues, providing a glimmer of hope.

## 4 Conclusions

Groundwater is the only source of water available to people in this study region for drinking and irrigation needs. For this reason, it is imperative that the hydrogeochemical studies in this area be carried out with the goal of determining the water quality for irrigation and drinking purposes. World Health Organization (WHO) data and hydrogeochemical results were compared. The salty water condition of the majority of samples was caused by the intrusion of seawater by hydrogeochemical processes. High amounts of sodium and calcium, along with chloride dominance, might have resulted either from anthropogenic activities or seawater incursion at various studied sites due to the proximity of each location to the coast. These water samples pH, EC, and TDS values were unsafe for drinking and irrigational use. In most regions, the concentrations of these water quality parameters were found unacceptable limits, such as sodium, potassium, calcium, magnesium chloride, carbonate, bicarbonate, sulfate, iron, and arsenic. The cation and anion dominant sequences in the research area's groundwater were found Na^+^> Ca^2+^ > Mg^2+^ > K^+^ and HCO_3_^−^ > Cl^−^ > SO_4_^2−^ > NO_3_^−^; Na^+^, Mg^2+^, K^+^, and Cl^−^ respectively, were the main causes of the TDS value. According to the Piper diagram, most of the water in the research areas was impacted by salty water. Few samples in the evaporation dominance zone and most in the rock dominance zone are seen in the Gibbs plots. Most groundwater found with high salinity, EC, Na%, SAR, RSC, TDS, and KR values suggests that they are unsuitable for irrigation purposes. To ensure safe agricultural activities and provide the general people with access to clean drinking water, sustainable management plans were required. In addition to minimizing the overuse of agrochemical pesticides and fertilizers, which caused the quality of the groundwater to decline, the study area requires an essential controlling mechanism. The success of water quality management for sustainable development is largely dependent on public awareness campaigns about the negative effects of poor water quality on human health, agricultural fields, and rainwater conservation techniques. This finding has the potential to significantly contribute to groundwater management initiatives in coastal areas of Bangladesh.

## Ethics approval and consent to participate

This study was conducted following the highest ethical standard. The data presented in this manuscript was accurate and authentic.

## Data availability statement

The data used to support the findings of this study are available from the corresponding author.

## Additional information

No additional information is available for this paper.

## CRediT authorship contribution statement

**A.H.M. Shofiul Islam Molla Jamal:** Writing – review & editing, Writing – original draft, Methodology, Investigation, Formal analysis, Data curation, Conceptualization. **Nisat Taslum Jhumur:** Investigation, Formal analysis, Data curation. **Md Aftab Ali Shaikh:** Writing – review & editing. **Mohammad Moniruzzaman:** Software, Formal analysis, Data curation. **Md Ripaj Uddin:** Data curation, Writing – review & editing, Investigation, Formal analysis, Data curation. **Md Abu Bakar Siddique:** Formal analysis, Data curation. **Muhammad Abdullah Al-Mansur:** Writing – review & editing. **Md Ahedul Akbor:** Formal analysis, Data curation. **Tajnin jahan:** Formal analysis, Data curation. **Sharmin Ahmed:** Formal analysis, Data curation, Formal analysis.

## Declaration of competing interest

The authors declare that they have no known competing financial interests or personal relationships that could have appeared to influence the work reported in this paper.
